# Retraction: Prediction of Mortality Based on Facial Characteristics

**DOI:** 10.3389/fnhum.2016.00515

**Published:** 2016-10-07

**Authors:** Frontiers Editorial Office

The journal retracts the 17 May 2016 article cited above. Following publication, concerns were raised regarding the scientific validity of the article. The Chief Editors subsequently concluded that aspects of the paper's findings and assertions were not sufficiently matched by the level of verifiable evidence presented. The retraction of the article was approved by the Chief Editors of Human Neuroscience and the Editor-in-Chief of Frontiers. The authors do not agree to the retraction.

